# Population Structure of Lassa Mammarenavirus in West Africa

**DOI:** 10.3390/v12040437

**Published:** 2020-04-13

**Authors:** Diego Forni, Manuela Sironi

**Affiliations:** Scientific Institute IRCCS E. MEDEA, Bioinformatics, 23842 Bosisio Parini, Italy; manuela.sironi@lanostrafamiglia.it

**Keywords:** Lassa mammarenavirus, geographic distribution, population structure, ancestry component, disease outcome

## Abstract

Lassa mammarenavirus (LASV) is the etiologic agent of Lassa fever. In endemic regions in West Africa, LASV genetic diversity tends to cluster by geographic area. Seven LASV lineages are recognized, but the role of viral genetic determinants on disease presentation in humans is uncertain. We investigated the geographic structure and distribution of LASV in West Africa. We found strong spatial clustering of LASV populations, with two major east–west and north–south diversity gradients. Analysis of ancestry components indicated that known LASV lineages diverged from an ancestral population that most likely circulated in Nigeria, although alternative locations, such as Togo, cannot be excluded. Extant sequences carrying the largest contribution of this ancestral population include the prototype Pinneo strain, the Togo isolates, and a few viruses isolated in Nigeria. The LASV populations that experienced the strongest drift circulate in Mali and the Ivory Coast. By focusing on sequences form a single LASV sublineage (IIg), we identified an ancestry component possibly associated with protection from a fatal disease outcome. Although the same ancestry component tends to associate with lower viral loads in plasma, the small sample size requires that these results are treated with extreme caution.

## 1. Introduction

Lassa mammarenavirus (LASV, family *Arenaviridae,* genus *Mammarenavirus)* is the etiologic agent of Lassa fever, a haemorrhagic disease endemic in parts of West Africa such as Nigeria and the Mano River Union (Guinea, Liberia, and Sierra Leone) (https://www.cdc.gov/vhf/lassa/index.html) [1]. Sporadic cases of Lassa fever have also been reported in Benin and Togo [2,3]. Lassa fever was first described in 1969 in the town of Lassa, Nigeria, where three missionary nurses became ill [4]. Among them was Lily Pinneo, after whom the prototype strain was named. 

Annually, an estimated 100,000 to 300,000 infections of Lassa fever occur, resulting in approximately 5000 deaths (https://www.cdc.gov/vhf/lassa/index.html). Although about 80% of people infected with LASV have mild or no symptoms, the case fatality rate among patients who are hospitalized with Lassa fever is around 15% (https://www.cdc.gov/vhf/lassa/index.html) [1]. 

Human-to-human transmission of LASV is rare and most infections are transmitted by rodents, via direct contact with infected animals or their excreta [5,6]. The main viral reservoir of LASV is the Natal multimammate mouse (*Mastomys natalensis*), although other rodents are increasingly recognized as alternative hosts for the virus [7,8]. Natal multimammate mice are widespread throughout Sub-Saharan Africa [9] and display a commensal behavior, often living and nesting in houses [10,11,12,13]. Despite the wide geographic range of the multimammate mouse, Lassa fever is confined to West Africa and it has never been reported east of Nigeria. In general, a relatively narrow geographic range seems to be a characteristic of most mammarenaviruses [6]. In fact, efforts to describe the genetic diversity of LASV in the endemic region indicated that viral genomes also tend to cluster by geographic area [6]. At least seven LASV lineages (and several sublineages) have been tentatively recognized. Lineages I, II, and III are transmitted in Nigeria, whereas lineage IV circulates in Liberia, Guinea, and Sierra Leone [14,15,16,17,18,19,20]. Strains from Mali and the Ivory Coast account for lineage V [21], and lineage VI includes a few Nigerian sequences, one isolated from an African wood mouse (Hylomyscus pamfi) and the others from Lassa fever patients [7,19]. Finally, two recently sampled sequences from Togo were proposed to represent the seventh lineage [2].

In line with the observation that LASV diversity is higher in Nigeria than elsewhere, phylogeographic analyses indicated that the virus originated in this country and moved westward to reach the Mano River Union and nearby countries [14,15]. Recent data have also provided information on the most likely geographic origin of LASV lineages II and III in the south-east and north of Nigeria, respectively. However, a detailed analysis of the spatial structuring of LASV populations in West Africa is missing. 

From a public health perspective, other relevant but missing information concerns the transmissibility and virulence in humans of the distinct viral lineages. To date, the majority of human-derived LASV sequences belong to lineages II and IV and case fatality rates were reported to be higher in Sierra Leone than in Nigeria [15]. It is, however, unclear whether such differences are the result of viral genetic determinants [15,22]. Another open question is why only a minority of subjects infected with LASV develop Lassa fever and, among those who do, what are the factors associated with disease outcome. Most likely, both viral and host factors play a role, but knowledge of either is presently poor [6,23,24].

Herein, we used different approaches to analyze the structure of LASV populations circulating in West Africa. We describe east–west and north–south gradients of viral genetic diversity and we suggest that LASV lineages diverged from an ancestral population that is now poorly represented among viruses sampled from humans. We also describe the structure of lineage II LASV populations and we report that a specific ancestry component might be associated with protection from severe disease. 

## 2. Materials and Methods

### 2.1. Sequences, Alignments, and Phylogenetic Trees

Complete or almost complete viral genomes were retrieved from GenBank and were previously sequenced by different groups to describe LASV diversity in Nigeria, Liberia, Guinea, Sierra Leone, Ivory Coast, Mali, and Togo [2,7,14,15,16,17,18,19,20,21]. Due to the over-representation of sequences from Edo State, Nigeria, a subset of genomes from this area were selected to be representative of sampling date. All strains sampled from rodents were included. A list of the 280 sequences and accession numbers is available in Table S1. 

Sequences of lineage IIg LASV with known disease outcome and plasma viral loads were obtained from GenBank and derive from previous works [15,17,18] (Table S2). 

Whole genome sequence alignments were generated using MAFFT [25] with default parameters. The L and S segment were separately aligned and concatenated. From complete genome alignments of 280 LASV sequences, 2569 biallelic parsimony-informative (PI) sites (sites that contain at least two types of nucleotides, each with a minimum frequency of two) were obtained. These sites were used as input for linkage disequilibrium (the nonrandom association of alleles along a chromosome or genome), population structure, and DAPC (discriminant analysis of principal components) analyses.

Phylogenetic trees were constructed using RAxML with 1000 bootstrap replicates [26].

### 2.2. Recombination 

Recombination was evaluated using four methods implemented in RDP4 (RDP, GENECONV, MaxChi, and Chimera) [27,28,29,30,31]. These methods were used because they showed good power in previous simulation analyses [30,32]. 

Only recombination events longer than 500 bp and detected by at least three methods (with a *p* value < 0.01) were considered as significant. RDP4 was run with general default parameters and no permutations, by setting sequences as linear, by requiring topological evidence, and by checking alignment consistency. For the four methods we applied, default parameters were also used (RDP: window size = 30; MaxChi: number of variable sites per window = 70, “strip gap” option on; Chimera: number of variable sites per window= 60; GENECONV: “treat indel blocks as one polymorphism” option on). 

### 2.3. Linkage Disequilibrium and Population Structure

Linkage disequilibrium (LD) was evaluated using the LIAN software v3.7 [33]. Briefly, LIAN tests for independent assortment by computing the number of loci at which each pair of haplotypes differs. Significance was assessed by Monte Carlo simulation (1000 iterations). As a measure of LD, LIAN computes a standardized index of association (I_A_S), which is zero for linkage equilibrium. 

We analyzed LASV population structure using the program STRUCTURE (version 2.3), which applies a Bayesian statistical model for clustering genotypes into populations without prior information on their genetic relatedness [34]. This method relies on a model in which the whole population is subdivided into K subpopulations characterized by a set of allele frequencies at each locus [34]. To run STRUCTURE, the allele frequency spectrum parameter (λ) was first estimated by using the “estimate λ” model for K = 1, as suggested [35]. We next applied the linkage model with correlated allele frequencies, which extends the admixture model to (weakly) linked loci [35]. This model has good power to detect subtle population structure [35]. To obtain more accurate inferences in spite of the different representation of genomes from distinct geographic areas, we used an ancestry prior that allows source populations to contribute deferentially to the pooled sample of individuals [36]. 

The admixture model with correlated frequencies also assumes that all subpopulations diverged from a common ancestral population, which is characterized by a set of allele frequencies estimated by the model. The amount of drift that each subpopulation experienced from these ancestral frequencies is quantified by the F parameter, which is thus similar in concept to the fixation index (F_ST_), a measure of population differentiation based on allele frequencies. 

To run STRUCTURE, map distances were set equal to PI site physical distances. The optimal number of populations was determined by running the model for different values of K. For each K, ten runs were performed with MCMC run lengths of 50,000 and 20,000 burn-in. Evanno’s method [37] and the trend of (LnPr(X|K), STRUCTURE documentation at https://web.stanford.edu/group/pritchardlab/structure_software/release_versions/v2.3.4/html/structure.html) [38] were used to select the optimal K with STRUCTURE HARVESTER [39]. Results of independent runs were merged by permutating clusters using CLUMPAK [40] to generate the Q-value matrix. To evaluate the contribution of the ancestral component to each PI site, a run with the optimal K was performed with the SITEBYSITE option selected.

### 2.4. DAPC Analysis 

A discriminant analysis of principal components, DAPC, [41] was applied using PIs as the input. DAPC was selected because it allows the identification of clusters of genetically related individuals. DAPC thus searches for synthetic variables (allele combinations) that maximize the variation among clusters while minimizing the variation within clusters. In DAPC, data are first transformed using PCA (to reduce the number of variables), the optimal number of clusters is identified, and then each sample is assigned to one of these clusters. The best number of clusters (K = 13) was identified through a Bayesian Information Criterion (BIC) (Figure S1), using a sequential K-means clustering method with K from 1 to 15. After that, a discriminant analysis of the number of principal components that explained more than 95% of variance was applied, and clusters of the first and second linear discriminants were plotted. DAPC was carried out with the Adegenet R package [42]. 

### 2.5. Statistical Analysis of Disease Outcome and Viral Loads

Logistic regressions were used to associate ancestry components (the variants in the genome that are derived from a specific parental population) to disease outcome. Because no information on the patients (e.g., sex, age) was available, no covariates were included in the models.

Viral loads were available for the two assays as RNA copies/ml. We only included patients with viral loads obtained from plasma. Viral loads were log-transformed and z-scores were calculated. The two assays (Altona and Nikisins) were combined by averaging z-scores. Differences were evaluated using *t*-tests (two-tailed). All calculations were performed in the R environment [43]. 

## 3. Results

### 3.1. Population Structure of LASV in West Africa

The program STRUCTURE is widely used to study population structure in relation to geographic origin. STRUCTURE can identify distinct subpopulations (or clusters, K) that compose the overall population and assigns each individual to one or more of those clusters. Subpopulations can then be related to specific features such as geographic origin, genotype classification, or phenotype. We thus considered that this approach might provide insight into the spatial distribution and ancestry of LASV genomes sampled in West Africa. Most LASV sequences available to date were collected in Edo state, Nigeria. Because sampling unevenness can bias STRUCTURE results [44], we excluded from the analyses a subset of genomes from this region. Conversely, all complete or almost complete LASV genomes sampled in other regions in Nigeria or outside the country were included, resulting in a final dataset of 280 sequences (Table S1).

Because STRUCTURE is ideally suited for weakly linked markers, we first analyzed the level of linkage disequilibrium with LIAN v3.7, which tests the null hypothesis of linkage equilibrium across loci [33]. Statistically significant LD was detected (Monte Carlo simulations, 1000 repetitions, *p* < 10^−3^), but the standardized index of association (I_A_S) was 0.063. This value, which indicates weak LD, is unlikely to be due to recombination, as this process is rare in mammarenaviruses [45]. Indeed, we searched for recombination events using RDP4 and we only detected seven events, which, in line with previous findings, derive from segment reassortment (Table S3). Therefore, as for other viral genome datasets [46], the weak association among sites is most likely due to the high mutation rates of LASV and/or to variable selective pressures across the genome. 

Whatever the underlying reasons, the weak LD we detected warrants the application of STRUCTURE models. Specifically, we used the linkage model with correlated allele frequencies, which assumes that discrete genome chunks are inherited from K ancestral populations [35]. As the representation of different geographic areas remains unbalanced, despite the exclusion of some sequences from Edo Sate, we ran STRUCTURE using an ancestry prior that allows source populations to contribute deferentially to the pooled sample of individuals [36]. This approach allows more accurate inference of the number of source populations and of individual ancestry proportions [36]. 

To estimate the optimal number of subpopulations in the LASV dataset, STRUCTURE was run for values of K from 1 to 12. The Δ*K* method yielded a major peak at K = 2 (Figure S2). However, Δ*K* is known to often underestimate K and to result in a value of 2 even when more subpopulations are present [38]. Because a second Δ*K* peak was evident for K = 10, which also corresponds to the plateau for the likelihood of the data (LnPr(X|K)) (Figure S2)*,* we considered that ten represents the most likely number of LASV source populations.

**Figure 1:**
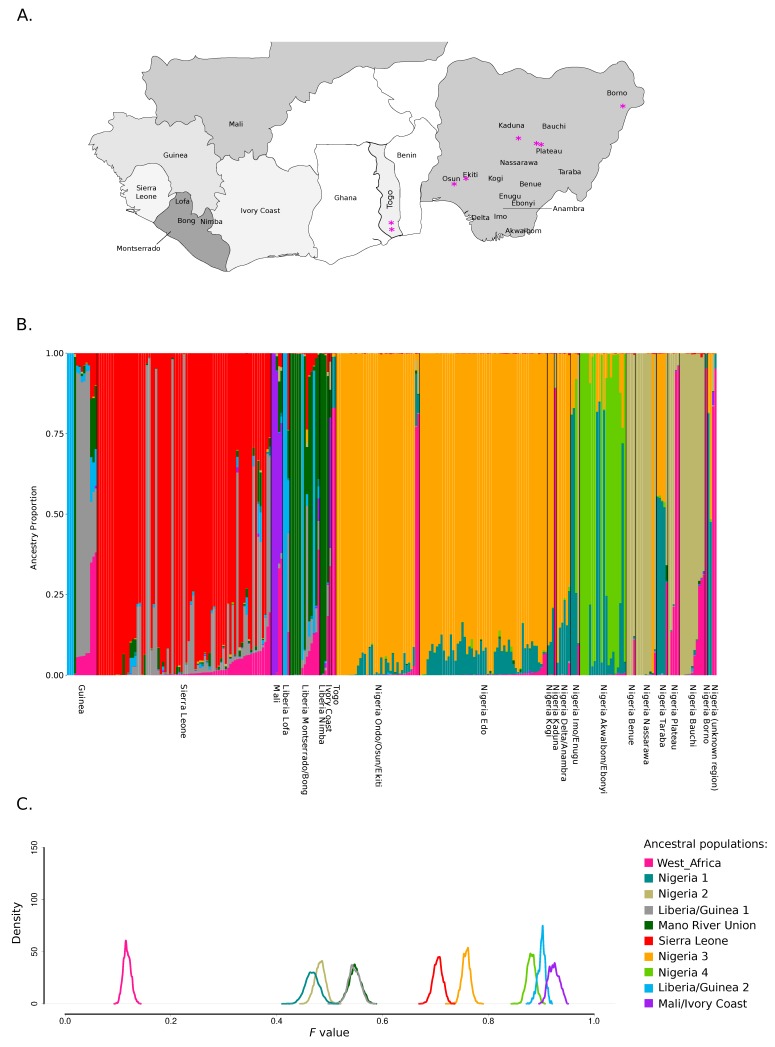
Population structure of Lassa mammarenavirus (LASV) in West Africa. (**A**). Map of the LASV endemic area in West Africa. Countries where LASV genomes were sampled are shaded in different hues of gray. In Nigeria and Liberia, the states/prefectures of origin of LASV sequences are shown. The magenta asterisks denote the origin of sequences having a major ancestry component from ancestral population West_Africa. (**B**). Bar plot representing the proportion of ancestral population components from the STRUCTURE linkage model for K = 10. Each vertical line represents a LASV genome. Genomes are ordered by country and by following a roughly west-to-east gradient. (**C**). Distributions of posterior *F* values for the ancestral populations. Colors are as in panel B.

Analysis of ancestry components was thus performed for K = 10 and by plotting genomes according to their geographic origin. Specifically, regions were ordered by country and by following a roughly west to east gradient (both among and within countries) (Figure 1A,B). Results showed a very good clustering by geography. Both within and outside Nigeria, most ancestral populations were specific for one or a few locations (e.g. ancestral population Sierra Leone, ancestral population Mali/Ivory coast, ancestral population Nigeria 2 for Northeastern Nigeria and ancestral population Nigeria 4 for Ebonyi state) (Figure 1B). The only exception was the ancestral population West_Africa, that was the major component of LASV genomes sampled in different areas of Nigeria and in the two Togo isolates; this population also contributed in varying proportion to genomes from all other countries. The population West_Africa represents the major component of the Pinneo strain, two additional archival samples isolated in Nigeria in 1981 and 1976, the divergent lineage VI sequence isolated from an African wood mouse (*Hylomyscus pamfi*), and a few recently sampled LASV Nigerian sequences from the Plateau and Kaduna states (Figure 1A,B) [16,19,20,47]. 

**Figure 2:**
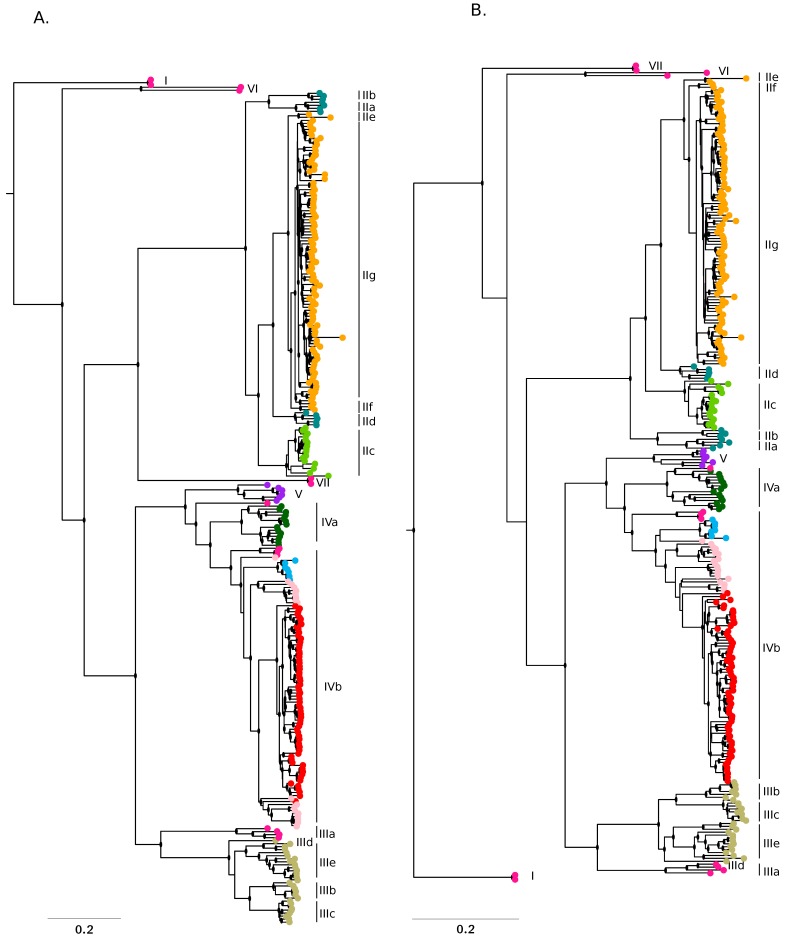
Phylogenetic trees. Phylogenies of the L (**A**) and S (**B**) segment were constructed using RAxML and the reference mobala virus sequence (L: NC_007904; S: NC_007903) as the outgroup. LASV sequences are colored according to the major ancestry component identified by STRUCTURE. Lineage and sublineage nomenclature is in accordance with a previous work [19]. Nodes with bootstrap support higher than 0.7 are denoted with a black dot.

The major ancestry components inferred by STRUCTURE broadly corresponded to the recognized LASV lineages (Figure 2A,B). The only exception was accounted for by sequences having a major ancestry component from ancestral population West_Africa: they did not cluster together and most of these sequences fell basal to the whole phylogeny or to specific lineages. We thus reasoned that this component might represent the most ancestral population from which LASV lineages diverged. 

To explore this possibility, we used the linkage model in STRUCTURE to estimate the level of drift of each subpopulation from a hypothetical common ancestral population. Specifically, we estimated the *F* parameter, which represents a measure of genetic differentiation between populations based on allele frequencies. Results indicated that population West_Africa had the lowest *F* value among all populations, indicating that it diverged little from the ancestral common population and possibly represents the ancestral source of known lineages (Figure 1C). Intermediate/low *F* values were also observed for ancestral populations Nigeria 1, Nigeria 2, Liberia/Guinea 1, and Mano River Union (Figure 1C). Sequences having the strongest representation of population Nigeria 1 (defining lineages IIa, IIb, and IIc) fall basal to all lineage II sequences and were mostly sampled in Ebonyi, Imo, and Enugu states [18,19] (Figure 2A,B); this result thus confirms previous data indicating that lineage II originated in this area [19]. Populations Mano River Union, and Liberia/Guinea 1 define genomes belonging to lineage IVa and a subset of IVb sequences, the remaining sequences of this latter clade being mostly found in Sierra Leone and having their ancestry from the corresponding population (Sierra Leone, with a higher *F* value) (Figure 1C, Figure 2A,B). Thus, these data are consistent with the introduction of LASV in Sierra Leone from Liberia or Guinea [15,20]. Finally, population Nigeria 2 is characteristic of lineages IIIe, IIIb, and IIIc, which circulate in the northeastern states of Nigeria (Figure 2A,B) [18,19]. The highest *F* values were observed for components found in lineage V genomes (from Mali and the Ivory Coast), in lineage IIc sequences (mostly from Ebonyi), and in a IVb subclade of viruses mainly sampled in an area of Guinea close to the Liberian border (Macenta) or in the flanking Lofa prefecture (Liberia) (Figure 1A,C and Figure 2A,B) [18,19,20,21]. With the exclusion of the Malian sequences, these genomes have limited diversity (Figure 1C and Figure 2A,B), suggesting that genetic drift was due to population bottlenecks and/or founder effects. In the case of sequences sampled in Mali and the Ivory Coast a nonrecent founder effect (with diversity recovery) is also likely, in line with the idea that LASV was introduced in these areas during colonial times [20,21].

### 3.2. Geographic Gradients of LASV Diversity

**Figure 3:**
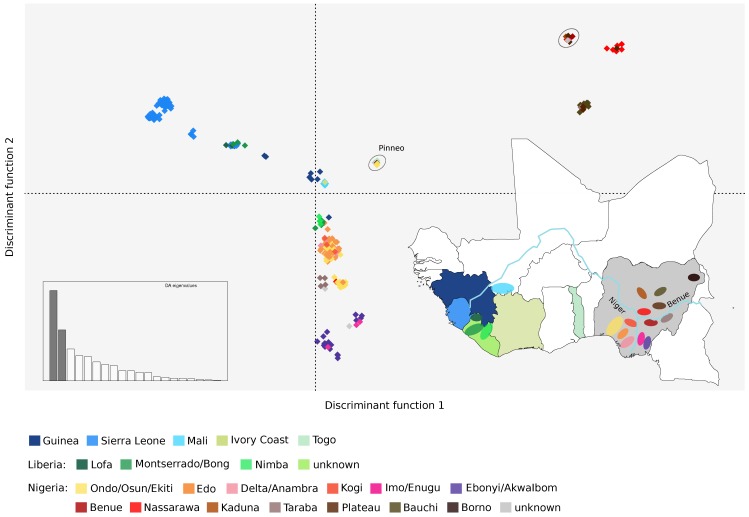
Geographic clustering of LASV genomes. Results of the discriminant analysis of principal components (DAPC) are shown. The first two discriminant functions, which explain the majority of variance in the data (inset), are plotted. Distance on the plot is proportional to genetic distance between clusters. Samples are color-coded based on their geographic origin, as shown in the map of West Africa. The two clusters containing sequences with a large proportion of ancestry from population West_Africa are circled (the one with the Pinneo strain is marked).

To further analyze the geospatial distribution of LASV genetic diversity, we applied a discriminant analysis of principal components (DAPC). This approach can identify the principal components that explain most between-group variation while minimizing within-group variation [41]. DAPC indicated the presence of 21 clusters, which showed very good correspondence to the geographic areas where viruses were sampled (Figure 3). Clusters were clearly separated by the two first discriminant functions along east–west and north–south axes. Within Nigeria, a clear separation was evident between viruses sampled east or west of the Niger river, as well as south or north of the Benue river (Figure 3). The only exceptions were the four sequences from Taraba state that clustered close to those sampled in Edo and Ondo, and the strains from Benue state that belonged to the same cluster as those collected in the northern regions (Figure 3). These results are in agreement with those obtained with STRUCTURE, whereby the Benue State LASV sequences had similar ancestry components as viruses sampled north of the Benue river, whereas sequences from Taraba showed a distinctive admixture pattern different from other strains (Figure 1B). Also in line with STRUCTURE results, several sequences with a high West_Africa ancestry component (the Pinneo strain, the Togo sequences, the archival sample of 1976, and the lineage VI genomes) formed a separate cluster, whereas the others belonged to a cluster with the Plateau sequences and the 1981 archival sample (Figure 3). 

### 3.3. Population Structure of LASV Lineage II and Association with Disease Severity

The majority of LASV genomes sequenced in Edo and Ondo States belong to lineage IIg. For 117 of these sequences, the clinical outcome of the infected patients (death or recovery) was also reported (Table S2) [15,17,18]. We thus wished to determine whether any substructuring was evident among lineage IIg LASV genomes and if any specific ancestry component was associated with clinical outcome. To this aim, we aligned sequences that were inferred to belong to clade IIg and with known clinical outcome. From the alignment, we extracted PI sites and we again found weak LD (I_A_S = 0.055). STRUCTURE was run for values of K from 1 to 10 and the ΔK method yielded a major peak at K = 5 (Figure S3). This latter value also roughly corresponds to the plateau of LnPr(X|K) (Figure S3), suggesting the presence of 5 clusters. 

**Figure 4:**
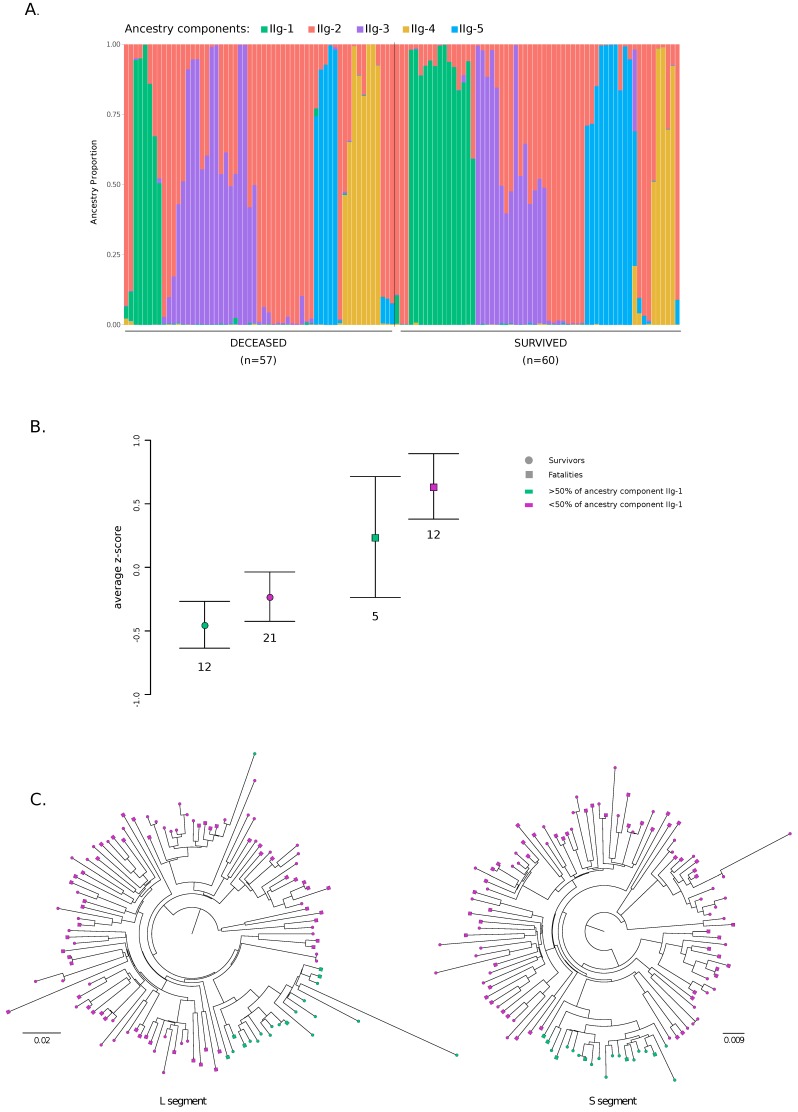
Population structure for lineage IIg and association with clinical outcome. (**A**). Bar plot representing the proportion of ancestral population components from the STRUCTURE linkage model for K = 5. Each vertical line represents a LASV genome. Genomes are ordered by clinical outcome. (**B**) Comparisons of plasma viral loads (average z-scores) for patients infected with viruses having more or less that 50% of ancestry from component IIg-1. Values are reported as mean and standard error. (**C**) Phylogenies of the L and S segment for lineage IIg viruses were constructed using RAxML. Tips are coded to indicate disease outcome. Genomes with more that 50% of ancestry from component IIg-1 are in green.

The representation of ancestry components was different between viruses that determined a fatal outcome and those that did not (Figure 4A). In particular, logistic regression indicated that ancestry component IIg-1 was significantly associated with recovery (*p* value = 0.047, OR = 0.76, 95% confidence intervals = 0.58–0.99), whereas no significant association was observed for the other components. 

Given these results, we wished to determine whether the IIg-1 component was also associated with lower viral loads. Plasma viral loads were available for 50 subjects, all of them recruited in 2018 and tested for LASV positivity using two different assays (the Altona and Nikisins RT-PCR assays, https://www.altona-diagnostics.com/; [48]) [18]. Viral loads obtained using the two assays were combined by averaging z-scores. As expected [49], patients who survived had lower viral loads than those who died of the infection (*t*-test, *p* = 0.0040). Patients infected with viruses having a major contribution (higher than 0.5) of ancestry component IIg-1 tended to have lower viral loads than those infected with other viruses (Figure 4B). This was evident for both survivors and nonsurvivors. Similar results were obtained when data from the two assays were analyzed separately (Figure S4). Possibly due to the small samples size, statistical significance was not reached, suggesting that these data should be taken with caution.

On phylogenetic trees of the L and S segment, LASV genomes with a major contribution (higher than 0.5) of ancestry component IIg-1 define a subclade of 20 sequences, 14 of which were sampled from subjects who survived the infection (Figure 4C). The site-by-site inference in STRUCTURE allows population-of-origin assignment for individual variants. Clearly, for genomes having a major proportion of their ancestry from component IIg-1, most variants along the genome had high probability (>0.9) to derive from this component. However, component IIg-1 tended to be evenly distributed along the L and S segments also for viruses with lower portions of this ancestry (Figure S5). Analysis of variants having > 0.9 probability of ancestry from component IIg-1 in all genomes indicated that 23 are synonymous and 7 nonsynonymous. Among nonsynonymous variants, 6 are located in the L protein (H359, N894, R1054, K1589, R1673, and I1679, numbering refers to the 2018/IRR_126 strain) and one is located in the C-terminal domain of NP (S485). These sites, and particularly the one in the RNA-dependent RNA polymerase (RdRp) domain (R1054) of L, represent good candidates as modulators of clinical severity, although validation in much larger patient cohorts will be necessary. 

## 4. Discussion

Detailed phylogenetic and phylogeographic analyses indicated that LASV most likely originated around 1000–2000 years ago in Nigeria [15,21], and that viral genetic diversity tends to cluster by geographic area [1,6]. Whereas most previous studies used phylogeographic inference and focused on distinct geographic areas [15,16,17,18,19,20], we applied a complementary approach to study the geographic structuring and distribution of LASV in the entire endemic zone. STRUCTURE and DAPC analyses are ideally suited to study geographic patterns of genetic diversity. One of the advantages of both methods is that they rely on clustering algorithms that infer the optimal number of populations (or clusters) and assign genomes to such populations probabilistically, without any prior specification about their relationships or geographic origin. As expected, we found strong spatial clustering of LASV populations with two major east–west and north–south diversity gradients. Population structure was also evident when the ancestry components of viruses sampled in different regions were analyzed. As noted previously [1,6], such effects are probably due to the limited dispersal range of rodent populations, which is constrained by natural barriers such as rivers and ridges [9,12,13]. Nonetheless, other factors, such as the preferential association of distinct viral lineages with specific rodent subtaxa, might also play a role [50]. 

By estimating the level of drift from a hypothetical common population, the STRUCTURE model we applied also allows inference about the most likely original location of the sampled genomes, as well as of migration events [35]. Overall, our data indicate that known LASV lineages diverged from an ancestral population most likely circulating in Nigeria, although alternative locations, such as Togo or nearby poorly sampled countries (i.e., Benin and Ghana) cannot be formally excluded. In fact, extant sequences carrying the largest contribution of this ancestral population include the prototype Pinneo strain (from Borno state), some archival samples, the Togo isolates, as well as viruses isolated after 2008 in different regions within Nigeria (i.e., the Kaduna, Osun, Ekiti, and Plateau states). It is noteworthy that viruses carrying this ancestral component are not commonly detected in LASV patients. One possibility is that the ancestral lineage was replaced by other lineages and is now uncommon in rodent populations. Alternatively, the ancestral component might be generally associated with no or mild disease presentation. However, because one of these sequences was isolated from an African wood mouse, the possibility that these viruses circulate in rodents that are not human commensals (and are therefore rarely transmitted) should not be dismissed. Interestingly, LASV was recently detected in pygmy mice (Mus Baoulei), another noncommensal species, in Benin [8]. The two partial LASV genomes showed closest similarity to the Togolese isolates, as well as to another unclassified virus, Jirandogo virus, which was also detected in pygmy mice in Ghana [51]. These observations might even suggest that the Natal multimammate mouse does not represent the ancestral LASV reservoir and that the switch to this host accounted for the marked drift of all other LASV populations from the common ancestral one. Clearly, additional genomes sampled from rodents will be necessary to disentangle these alternative scenarios. 

The ancestry component showing lowest drift is also the only one detectable both within and outside Nigeria, supporting a single, nonrecent introduction of LASV in the Mano River Union [14,15]. It was previously suggested that LASV migrated westward from Nigeria to reach the Ivory Coast, Guinea, Liberia, and finally Sierra Leone [15]. More recently, the sequencing of additional LASV genomes from Liberia suggested that this country represented the entry point of LASV in the Mano River Union [20]. The methods we applied are not devised to directly infer the direction of migration events. However, both STRUCTURE and DAPC analyses are consistent with the introduction of LASV in Sierra Leone from either Liberia or Guinea. Concerning the Ivory Coast, the availability of a single genome from this region makes inferences about its role as a passage route for LASV quite weak, at least using the methods we applied. In any case, as previously suggested, the spatial distribution and divergence of LASV lineages is unlikely to only result from autonomous rodent movements, as human-mediated transportation of commensal and peri-commensal species likely played a role [20,21]. For instance lineage V was previously suggested to have reached Mali via human movement during the colonial period [20,21]. However, the timing of separation of this lineage from the common ancestor with lineage IV [20] might also suggest that contacts among local populations earlier than colonial time introduced LASV to Mali. Both scenarios are in line with our results showing genetic diversity but strong drift of this lineage, suggesting a founder effect with subsequent population expansion. 

It remains presently unknown whether the divergence of LASV lineages and sublineages was accompanied by changes in terms of human transmissibility and disease presentation or severity. This issue is not easily addressed for different reasons. First, disease presentation most likely results from the interplay of viral and host factors (e.g., genetic background, age) [23,24]. Second, most LASV genomes were obtained from patients who developed Lassa fever and the mild/asymptomatic cases remain unsampled. Third, most clinical samples derive from patients infected with lineages II and IV, making comparison among lineages very difficult. Moreover, because different lineages are transmitted in different geographic areas, disparity in time to diagnosis and treatment might influence disease outcome. Finally, experimental infection of rodent models with other mammarenaviruses have indicated that subtle differences among strains can drastically affect disease presentation, suggesting that virulence determinants might segregate at the level of subclades or small sequence clusters [52,53,54,55,56,57,58,59]. For instance, a cluster of high case fatality rates in Taraba state was recently reported [24]. We thus decided to assess the association between disease outcome and viral genome by focusing on sequences from a single sublineage (IIg), which represent the majority of clinical samples available to date. All these sequences were obtained from patients from Edo or Ondo sates who were hospitalized at the Irrua Specialist Teaching Hospital (ISTH) [15,17,18], suggesting similar treatment options. We found that one ancestry component is associated with protection from a fatal outcome of LASV infection. The same ancestry component tended to associate with lower viral loads in plasma, irrespective of the outcome. However, we warn that the statistical significance of these findings is borderline or not reached. Moreover, the differences were based on a small sample of infected subjects, and viruses having a substantial proportion of IIg-1 ancestry were a minority. Also, the case fatality rate of Lassa fever increases with age [24], which should thus be incorporated in statistical models of genetic association. These considerations imply that our results concerning a protective role of ancestry component IIg-1 should be treated with extreme caution and should be merely considered as a starting point for future follow-up analyses. 

## Supplementary Materials

The following are available online: https://www.mdpi.com/1999-4915/12/4/437/s1. Table S1. list of 280 strains used for STRUCTURE analysis and DAPC; Table S2. list of lineage IIg strains with outcome information; Table S3. recombination events; Figure S1. Population structure of LASV in West Africa: analysis of optimal K; Figure S2. DAPC cluster selection; Figure S3. Population structure of lineage IIg: analysis of optimal K; Figure S4. Plasma viral loads in clade IIg strains; Figure S5. Variant distribution of IIg-1 component.
