# Supplementary material for: Population Structure of Lassa Mammarenavirus in West Africa
Source: Viruses. 2020 Apr 13;12(4):437. doi: 10.3390/v12040437 (PMC7232344; doi:10.3390/v12040437)
Supplement: Supplementary file 1 [file viruses-12-00437-s001.zip › Supplementary_Material_def.pdf]

Supplementary Material

Figure S1. DAPC cluster selection. Bayesian Information Criterion (BIC) values versus number of clusters.

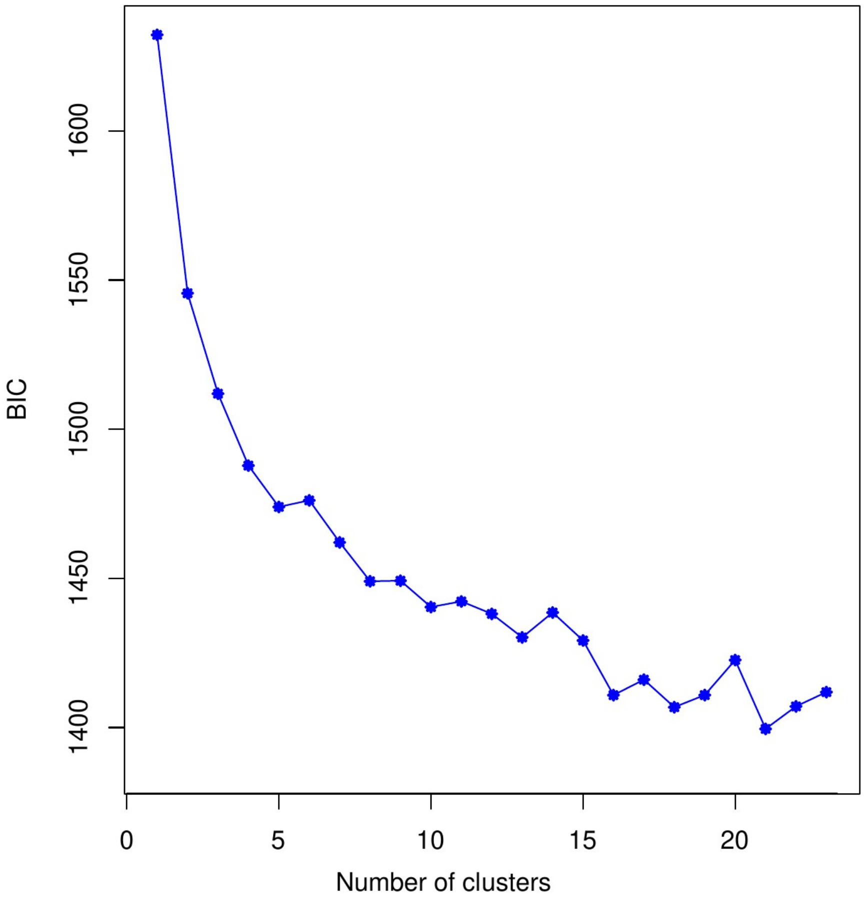

Figure S2. Population structure of LASV in West Africa. Analysis of optimal K for STRUCTURE analysis. **A.** Mean and variance of ln(Pr(X|K) for K values from 1 to 12. **B.** ΔK for values of K from 2 to 11. ΔK is calculated as ΔK = mean(|L"(K)|) / sd(L(K)). Plots were generated with STRUCTURE HARVESTER [1].

A.

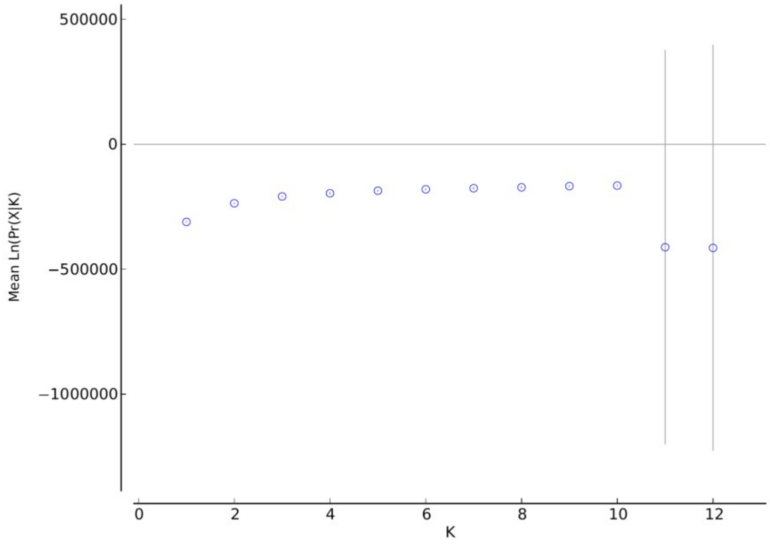

B.

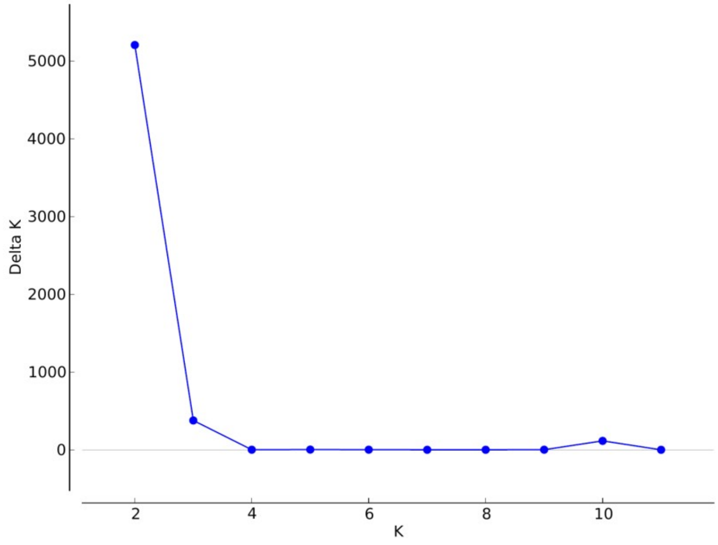

Figure S3. Population structure of lineage IIg. Analysis of optimal K for STRUCTURE analysis. **A.** Mean and variance of ln(Pr(X|K) for K values from 1 to 10. **B.** ΔK for values of K from 2 to 9. ΔK is calculated as ΔK = mean(|L"(K)|) / sd(L(K)). STRUCTURE HARVESTER [1].

A.

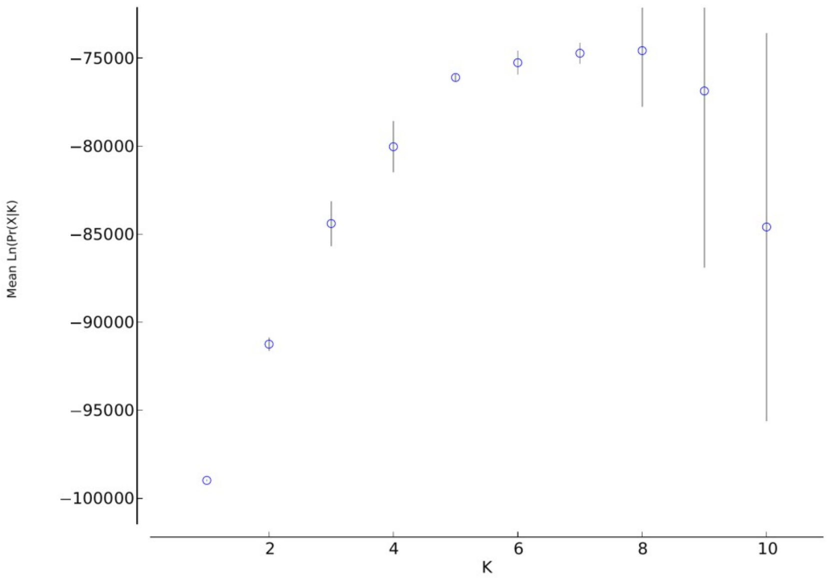

B.

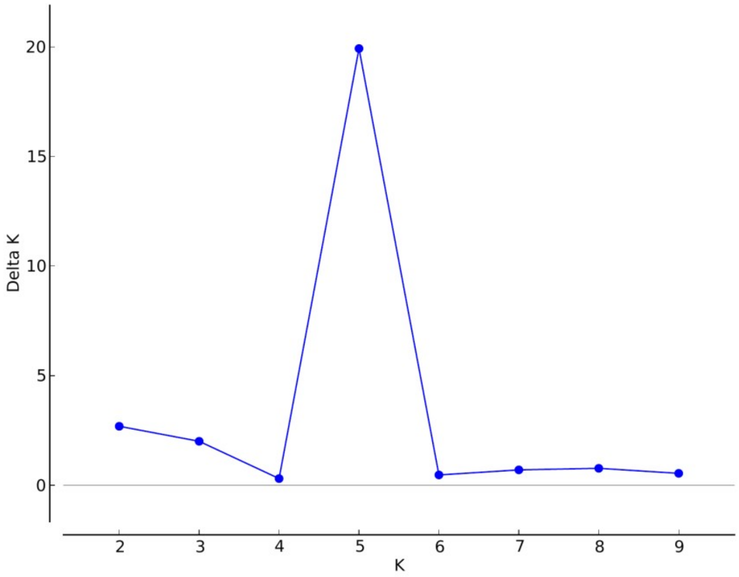

Figure S4. Plasma viral loads in clade IIg strains. Comparisons of plasma viral loads (z-scores) for patients infected with viruses having more or less that 50% of ancestry from component IIg-1. Values are reported as mean and standard error. The asterisk denotes a significant difference (t-test, two-tailed, p= 0.05).

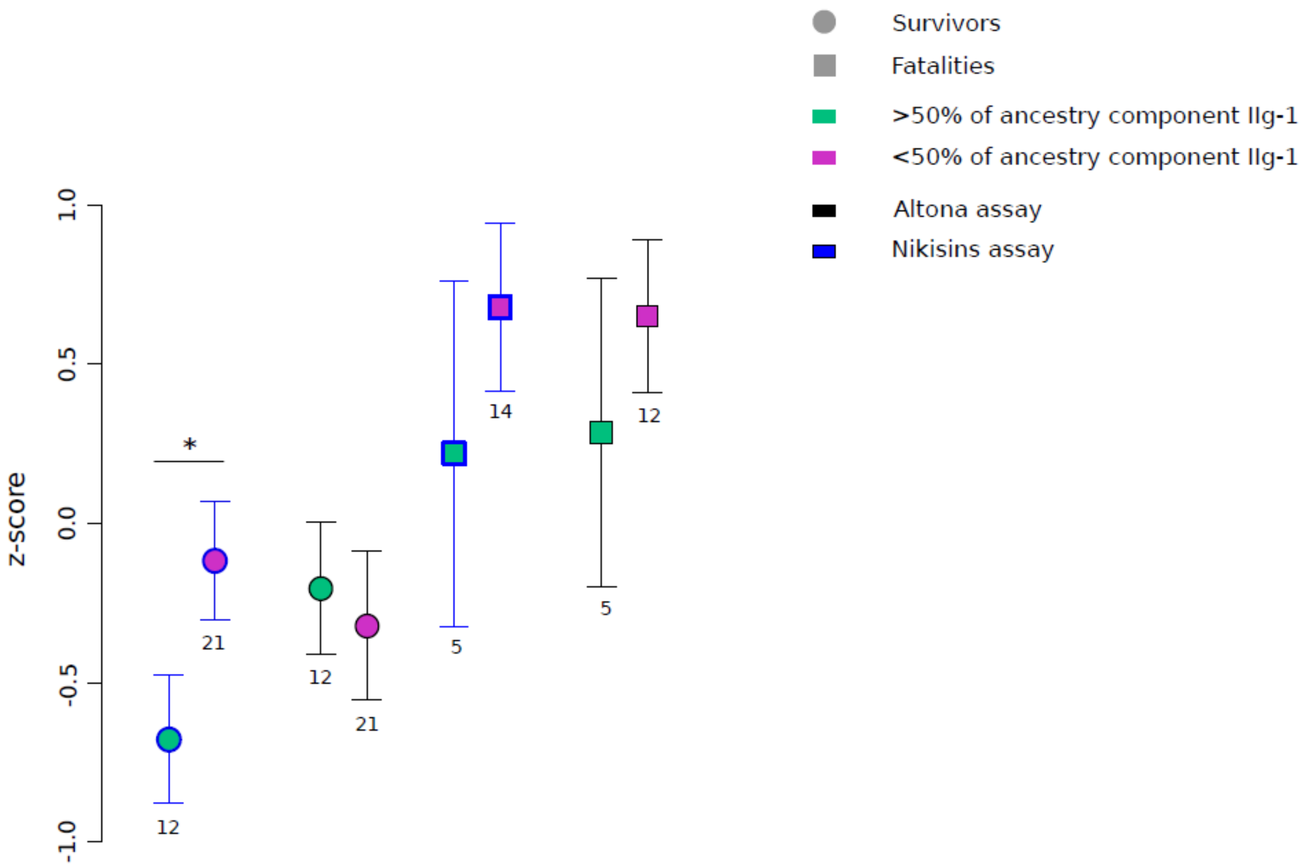

Figure S5. Variant distribution of IIg-1 component. Site by site probability for a few representative II-g strains having at least 50% of ancestry component IIg-1. Each line represents a genome and each dot represents a SNP with a probability >0.90 to derive from the component IIg-1. A schematic representation of a LASV genome is also shown.

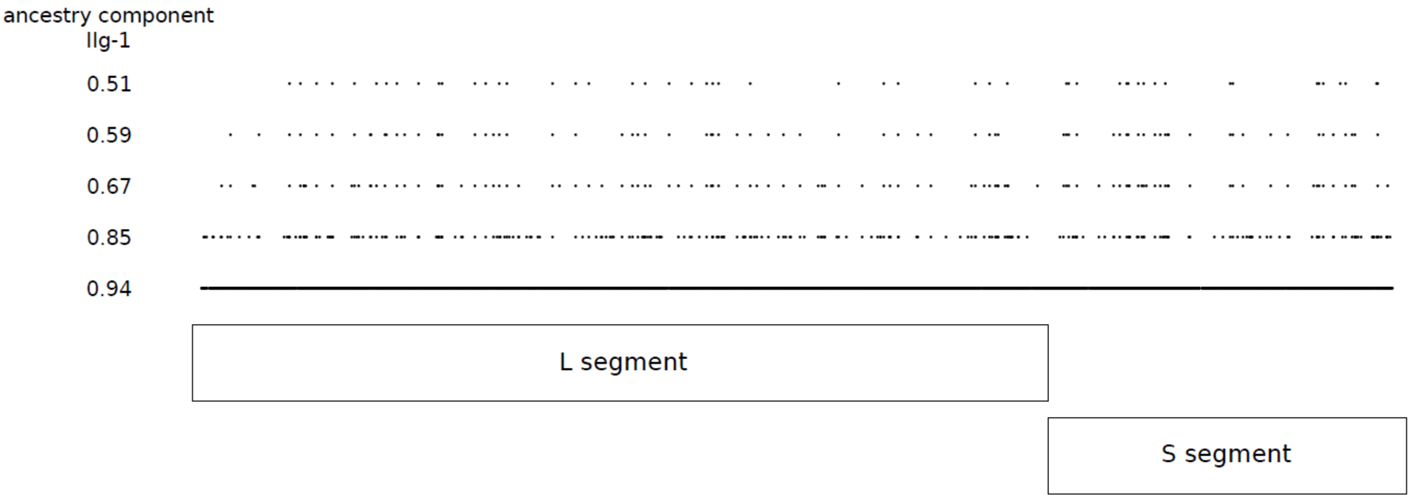

Table S3. Recombination events detected with RDP4.

Event	Begin	End	Recombinant	Minor Parental	Major Parental	Notes	Detection Methods (p values)			
							**RDP**	**GENECONV**	**Maxchi**	**Chimaera**
1	8214	12232	LM774-SLE-2012	LM778-SLE-2012	LM776-SLE-2012	Already described [2]	4.18E-19	6.58E-18	1.65E-09	4.15E-10
2	8509	12165	G2363-SLE-2012*	G1529-SLE-2011	Unknown		1.16E-18	NS	1.16E-08	1.09E-06
3	8582	12150	LM779-SLE-2012	G692-SLE-2009	G502-SLE-2009		1.31E-15	NS	1.04E-08	2.16E-10
4	7838	12165	807978-LBR-1981*	LF17019	Unknown	Already described [3]	1.05E-14	NS	1.28E-04	7.80E-05
5	8300	11649	LASV/NGA/2018/IRR_091	LASV/NGA/2018/IRR_023	LASV/NGA/2018/IRR_036		3.08E-03	NS	1.54E-11	6.10E-11
6	7868	12161	LASV/NGA/2018/IRR_052	LASV/NGA/2015/IRR_011	LASV/NGA/2018/IRR_048		6.67E-09	NS	5.49E-07	1.07E-08
7	8280	12075	LASV/NGA/2018/IRR_075	ISTH2016-NIG-2012	Nig08-A41		5.94E-05	NS	1.77E-05	2.07E-06

NOTES: positions refer to genomic alignment
* Admixed in STRUCTURE analysis

References

1. Earl, D.A.; vonHoldt, B.M. STRUCTURE HARVESTER: A Website and Program for Visualizing STRUCTURE Output and Implementing the Evanno Method. *Cons Genet Res* **2012**, *4*, 359-361.

2. Andersen, K.G.; Shapiro, B.J.; Matranga, C.B.; Sealfon, R.; Lin, A.E.; Moses, L.M.; Folarin, O.A.; Goba, A.; Odia, I.; Ehiane, P.E.*; et al.* Clinical Sequencing Uncovers Origins and Evolution of Lassa Virus. *Cell* **2015**, *162*, 738-750.

3. Wiley, M.R.; Fakoli, L.; Letizia, A.G.; Welch, S.R.; Ladner, J.T.; Prieto, K.; Reyes, D.; Espy, N.; Chitty, J.A.; Pratt, C.B.*; et al.* Lassa Virus Circulating in Liberia: A Retrospective Genomic Characterisation. *Lancet Infect. Dis.* **2019**, *19*, 1371-1378
